# Evaluation of an artificial vertebral body fabricated by a tantalum-coated porous titanium scaffold for lumbar vertebral defect repair in rabbits

**DOI:** 10.1038/s41598-018-27182-x

**Published:** 2018-06-12

**Authors:** Faqi Wang, Lin Wang, Yafei Feng, Xiaojiang Yang, Zhensheng Ma, Lei Shi, Xiangyu Ma, Jian Wang, Tiancheng Ma, Zhao Yang, Xinxin Wen, Yang Zhang, Wei Lei

**Affiliations:** 1 Institute of Orthopaedics, Xijing Hospital, The Fourth Military Medical University, Xi’an, China; 2 Department of orthopedic surgery, the First Affiliated Hospital of Xi’an Jiaotong University, Xi’an, China; 3 The 463 hospital of Chinese Peoples’ Liberation Army, Shenyang, China; 4 The company of 31681 army, Tianshui, China

## Abstract

Tantalum (Ta)-coated porous Ti-6A1-4V scaffolds have better bioactivity than Ti-6A1-4V scaffolds; however, their bioperformance as an artificial vertebral body (AVB) is unknown. In the present study, we combined a Ta-coated Ti-6A1-4V scaffold with rabbit bone marrow stromal cells (BMSCs) for tissue-engineered AVB (TEAVB) construction and evaluated the healing and fusion efficacy of this scaffold in lumbar vertebral defects after corpectomy in rabbits. The results showed that BMSCs on the surface of the Ta-coated Ti scaffolds proliferated better than BMSCs on Ti scaffolds. Histomorphometry showed better bone formation when using Ta-coated TEAVBs than that with Ti TEAVBs at both 8 and 12 weeks after implantation. In addition, the vertical and rotational stiffness results showed that, compared with uncoated TEAVBs, Ta-coated TEAVBs enhanced rabbit lumbar vertebral defect repair. Our findings demonstrate that Ta-coated TEAVBs have better healing and fusion efficacy than Ti TEAVBs in rabbit lumbar vertebral defects, which indicates their good prospects for clinical application.

## Introduction

Vertebral corpectomy and anterior spinal decompression is a major surgical method for spine diseases, including spinal tumors, spinal tuberculosis and unstable burst vertebral fracture, which occasionally cause severe vertebral body destruction and result in neural impingement and spinal instability^1–3^. After this procedure, it is mandatory to reconstruct the vertebral defect. Autogenous bone, such as that from the ilium or fibula, is one alternative^4,5^. However, anterior autogenous bone has many disadvantages, including insufficient autologous bone mass and inability to provide sufficient immediate spinal stability postoperatively^6^. Titanium (Ti) mesh cages and artificial vertebral bodies (AVBs) are types of effective vertebral replacements that have been used in the clinic^7,8^. However, the currently used Ti mesh cages and AVBs have no effects of osteoconductivity, osteoinductivity, or osteogenesis, and under the premise of restoring spinal stability, they cannot promote the fusion of adjacent spinal segments^9^. Moreover, because of the difference in elastic modulus between Ti mesh cages or AVBs and vertebrae, long-term implantation may lead to complications such as bone stress concentration and bone absorption, causing instability or sinking of Ti mesh cages and AVBs or increasing the risk of adjacent spinal segment fracture, especially in osteoporosis (OP) patients^10^.

Tissue engineering that integrates engineering and life science is an emerging technology and is used to construct bioactive implants *in vitro*, some of which have been applied in the clinic. Osteoblastic differentiated bone marrow stromal cells (BMSCs) can promote osteogenesis on scaffolds and stimulate bone regeneration^11^. Electron beam melting (EBM) is a new additive manufacturing technique that has been successful in fabricating dense and porous Ti6Al4V scaffolds with better control of the internal structure and external shape^12–14^. In our previous work, we confirmed that the scaffold fabricated by EBM had similar biomechanics and elastic moduli to those of cortical bone *in vitro*^15^. The porous structure of the scaffold we designed had a honeycomb-like structure with a porosity of approximately 60 percent^16^, which could provide a proper scaffold for vascular ingrowth and new bone formation to provide an osteoconductive surface. However, pure porous Ti-6A1-4V scaffolds have limited biocompatibility and are unfavorable for the proliferation and differentiation of BMSCs, which may lead to insufficient osseointegration and repair effects in local bone defects^17^.

Recently, tantalum (Ta) has been used in orthopedic implants, such as arthroplasty prostheses and bone grafts, as a new metallic biomaterial and achieved good clinical results, with resistance to corrosion bioactivity *in vivo*^18^. The porous Ta can provide a scaffold for bone mechanical attachment and ingrowth^19–23^. Ta is a superior promoter of osteoinductivity^24^, and it offers a low modulus of elasticity and excellent bioactivity, biocompatibility and in-growth properties^25,26^. However, because of the inability to produce it modularly and the relatively high cost and lower fatigue resistance compared with fully dense materials, the utility of Ta implants is limited^27^.

Our previous work produced a Ta-coated porous Ti-6A1-4V scaffold and confirmed that its bioactivity was better than that of Ti-6A1-4V scaffolds^28^. The Ti scaffolds exhibited a Young’s modulus of 4.25 GPa, as previously described^16^, which is similar to that of human bone, thereby avoiding the mismatched elastic modulus. We assume that with a proper porous scaffold structure, Ta-coated surface modification and cultured BMSCs, the unsatisfactory osteoconductivity, osteoinductivity, or osteogenesis of clinical implants could be resolved. Therefore, the objective of this study was to fabricate a Ta-coated Ti-6A1-4V scaffold with a controlled porous structure using EBM and chemical vapor deposition (CVD) to construct a tissue-engineered AVB (TEAVB) combined with rabbit BMSCs and to evaluate the healing and fusion efficacy of TEAVB in a large lumbar vertebral defect after corpectomy in a rabbit model. This is the first study to use a Ta-coated Ti-6A1-4V TEAVB for the repair of intact vertebral defects.

## Results

### Morphological observation of the scaffold

**Figure 1:**
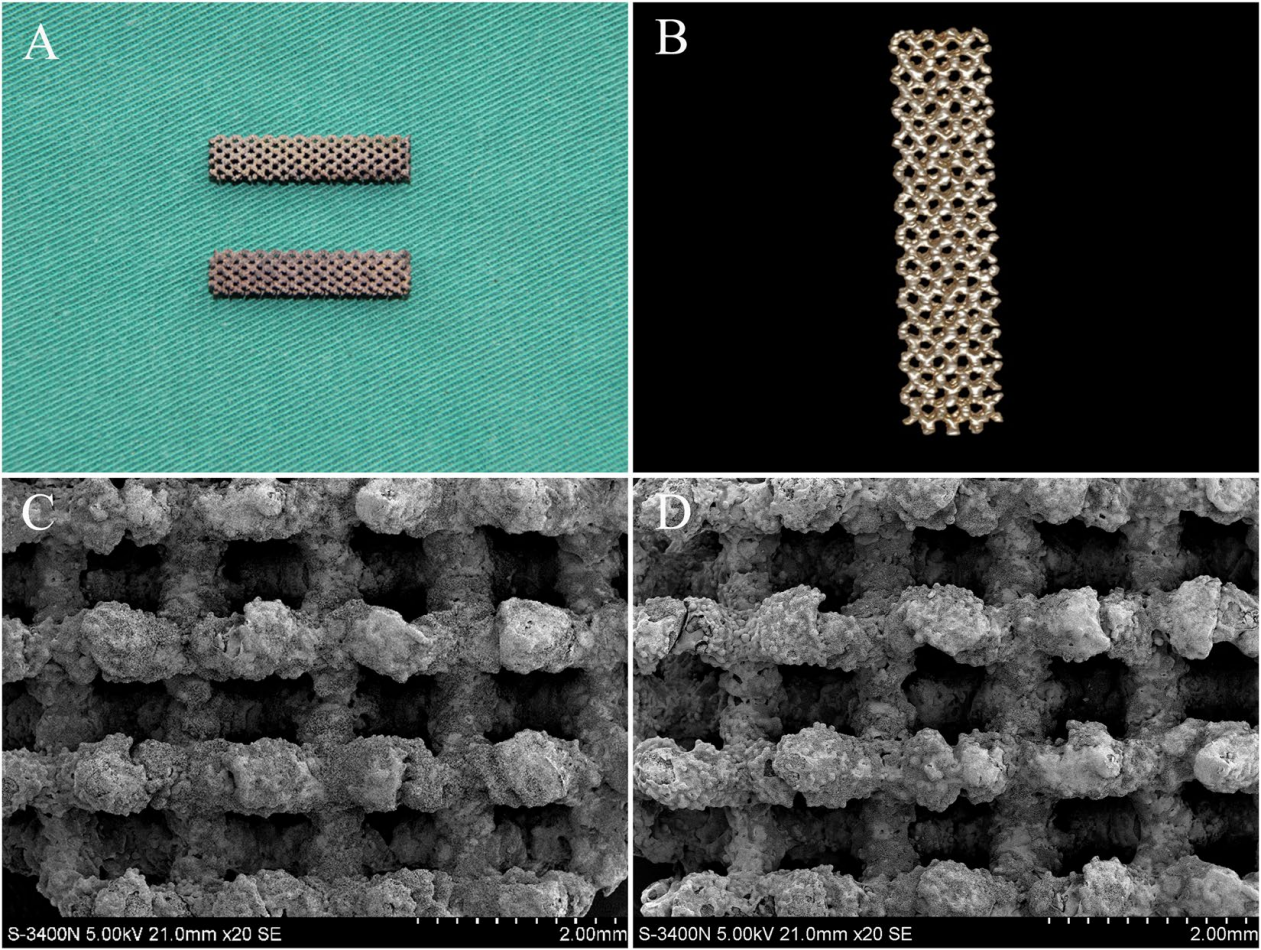
Porous Ti6Al4V scaffolds with and without Ta coating. (**A**) The general view of the scaffolds. Top: Ti6Al4V scaffold; bottom: Ta-coated Ti6Al4V scaffold. (**B**) Characteristic micro-CT image of uncoated and Ta-coated scaffolds. (**C,D**) The morphological structures of uncoated and Ta-coated scaffolds, respectively.

The porous Ti-6A1-4V scaffold vector with a diamond molecular structure was made with direct metal rapid prototyping (RP) technology. The scaffold had the color of Ta (gray), with a relatively dark luster after coating. The surface morphology showed that Ta had been deposited on the surface of pores in the scaffolds. Both pore structures and gap sizes of the porous Ti-6A1-4V scaffolds were basically consistent between pre- and postcoating, implying the favorable characteristics of the interconnected pore structure, which could facilitate cell adhesion, cell proliferation, tissue ingrowth, and the revascularization of scaffold vectors during defect repair (Fig. 1).

### Cell adhesion and morphology on the scaffold

**Figure 2:**
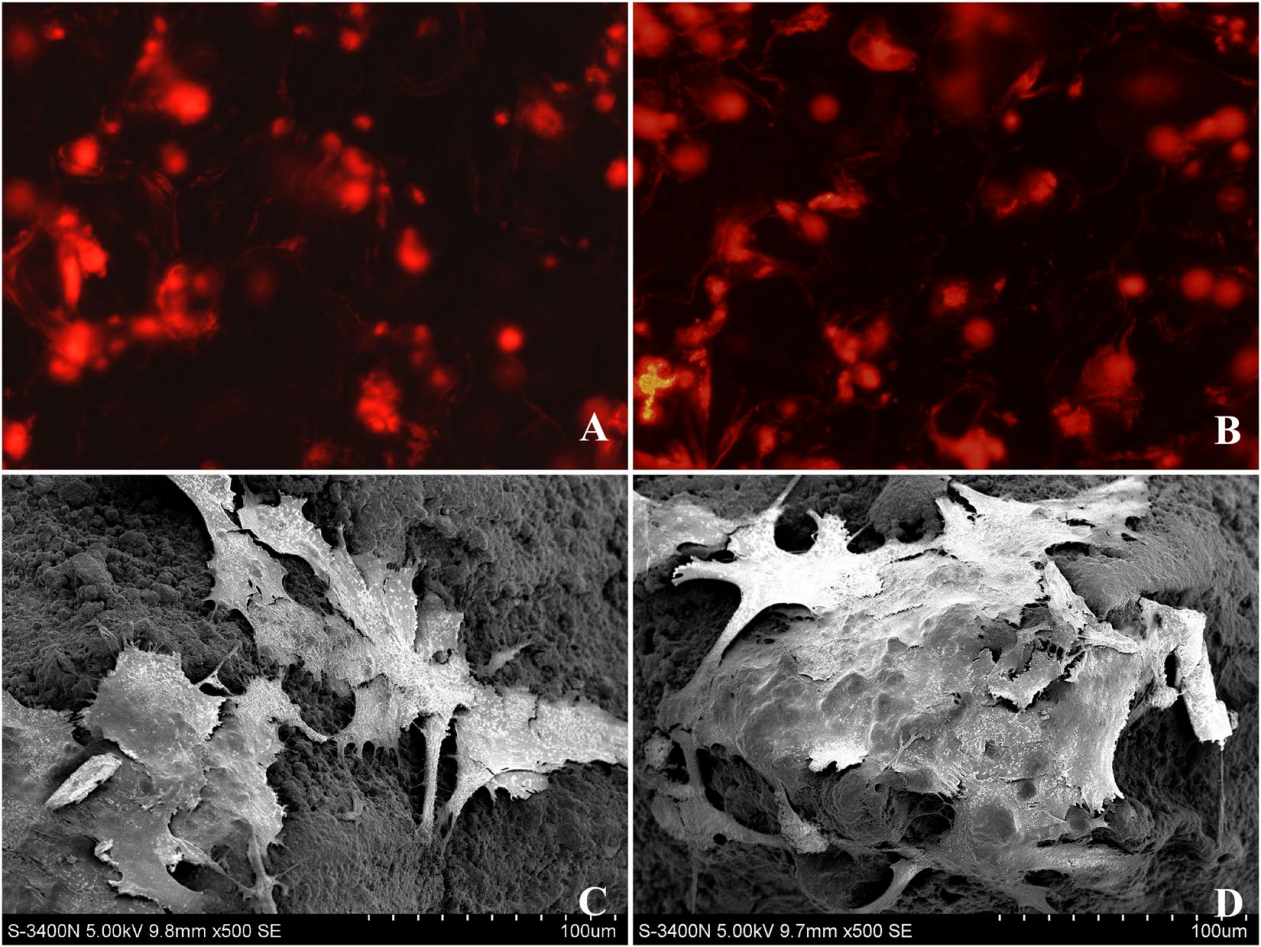
Morphological evaluation of BMSCs on the scaffolds. CM-DiI-labeled BMSCs attached to uncoated (**A**) and Ta-coated scaffolds (**B**) and SEM morphology of BMSCs attached to uncoated (**C**) and Ta-coated scaffolds (**D**) after 72 h of culture.

Fluorescence microscopy revealed that the CM-DiI-labeled BMSCs had a red color and clear morphology. The pseudopods extended completely and adhered closely to the surface of the scaffold vectors, while the nucleolus was colorless. More BMSCs adhered to the surface of Ta-coated scaffolds, indicating that they were favorable for cell adhesion and proliferation (Fig. 2A,B).

SEMs clearly showed the cell morphology and cell count on the surface of the two types of scaffolds after *in vitro* culture for 72 h. BMSCs had grains of secretory cell factors on the surface, and the protruded pseudopods were closely bound to the scaffolds. On the surface of the Ta-coated scaffolds, pseudopods were bound more closely to the scaffolds, and some pseudopods probed into the Ta micropores. In contrast, on the Ti scaffolds, the BMSCs were fusiform or streak-shaped with fewer pseudopods. BMSCs grew well on the surface of both scaffolds, without cell contraction or folding (Fig. 2C,D).

### Cell attachment, proliferation and ALP activity

**Figure 3:**
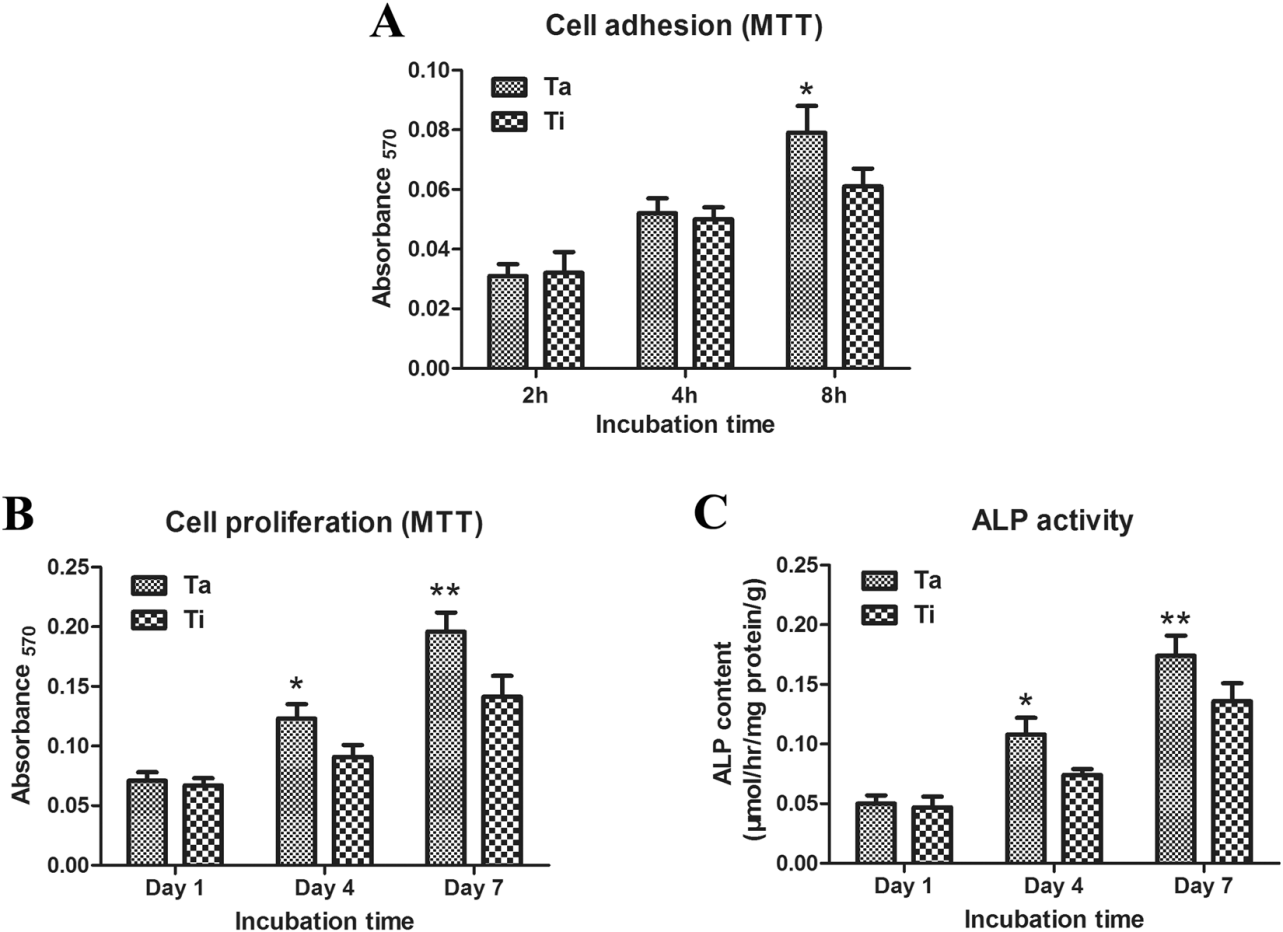
Attachment, proliferation and ALP activity of BMSCs on the scaffolds. (**A**) Adhesion of BMSCs cultured on uncoated and Ta-coated scaffolds at 2, 4, and 8 h. (**B**) Proliferation of BMSCs cultured on uncoated and Ta-coated scaffolds at 1, 4, and 7 d. (**C**) ALP activity of BMSCs cultured on uncoated and Ta-coated scaffolds at 1, 4, and 7 d. All data are expressed as the mean ± SD. *P < 0.05, **P < 0.01.

By MTT analysis, we found that although the counts of BMSCs adhering to the Ta-coated and Ti scaffolds were not significantly different at the cell culture time of 2 or 4 h, a longer cell culture period of 4 h successfully showed more adhering cells in the Ta-coated group than those in the Ti group (P < 0.05) (Fig. 3A). Moreover, BMSCs continued to grow on the surface of the two types of scaffolds when the cell culture time was further prolonged, and the cell counts were significantly higher on the Ta-coated scaffolds compared with those on the Ti scaffolds at both 4 and 7 d (P < 0.05) (Fig. 3B). In addition, the alkaline phosphatase (ALP) activity on the Ta-coated scaffolds was higher than that on the Ti scaffolds at both 4 and 7 d (P < 0.05) (Fig. 3C).

### Micro-CT analysis

**Figure 4:**
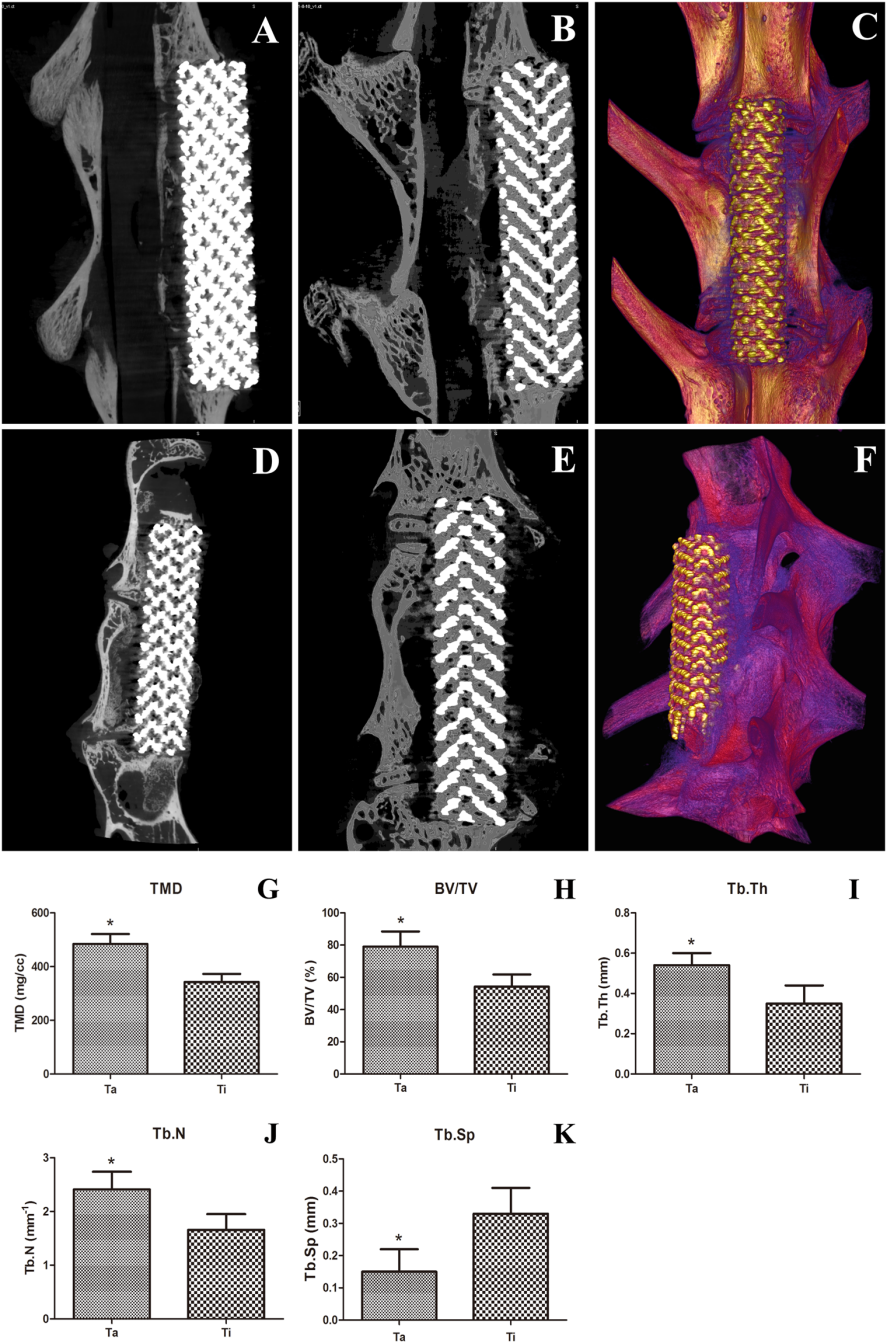
Micro-CT 3D reconstruction and bone tissue metrology analysis of scaffolds postoperation. (**A,D**) Micro-CT images of Ta-coated TEAVBs and Ti TEAVBs at 8 w, respectively. (**B,E**) Micro-CT images of Ta-coated TEAVBs and Ti TEAVBs at 12 w, respectively. (**C,F**) Micro-CT 3D reconstruction images of Ta-coated TEAVBs and Ti TEAVBs at 12 w, respectively. (**G,K**) Analysis of tissue mass density, bone volume fraction, number of bone trabeculae, thickness of bone trabeculae, and bone trabecular space at 12 w, respectively. All data are expressed as the mean ± SD. *P < 0.05.

Micro-CT 3D reconstruction showed that at the cell culture time of 8 w, there was little newly formed bone tissue around both scaffold types. However, there was more spongy bone between the Ta-coated TEAVBs and host bone compared with that between the Ti TEAVBs and host bone (Fig. 4A,D). At 12 w, synostosis formed between the Ta-coated TEAVBs and host bone, and the newly formed callus extended to the middle part of the defects, leading to more new bone mass compared with that resulting from the Ti TEAVBs (Fig. 4B,C,E,F). Bone tissue metrology showed that at 12 w, tissue mass density (TMD), bone volume fraction (BV/TV), number of bone trabecula (Tb.N) and thickness of bone trabeculae (Tb.Th) inside, on the surface, and around the Ta-coated TEAVBs were all significantly higher (P < 0.01), while the space of trabecular bone (Tb.Sp) was significantly smaller (P < 0.05) than those in the uncoated TEAVBs (Fig. 4G,K).

### Histological observation

**Figure 5:**
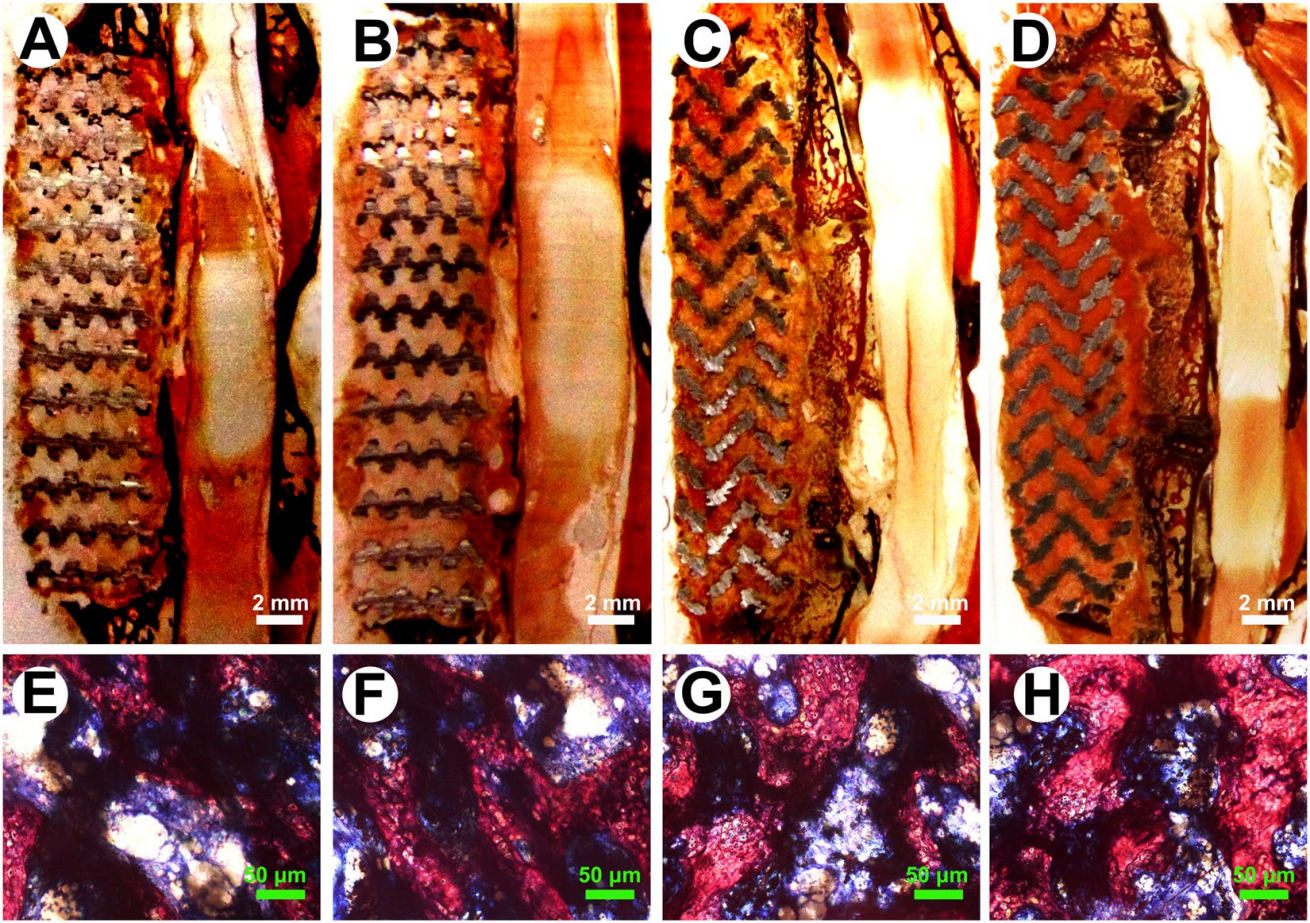
Representative histological analysis of scaffolds with VG staining postoperation in the L6 of rabbits. (**A,C**) General view of longitudinal sections of uncoated TEAVBs at 8 and 12 w of cell culture postoperatively. (**E,G**) Representative images of uncoated, VG-stained TEAVBs at 8 and 12 w of cell culture postoperatively. (**B,D**) General view of longitudinal sections of Ta-coated TEAVBs at 8 and 12 w of cell culture postoperatively. (**F,H**) Representative images of VG-stained, Ta-coated TEAVBs at 8 and 12 w of cell culture postoperatively. The tissue stained red was the newly formed bone with visible cell nuclei. The tissue stained blue was fibrous tissue. Scale bar: 2 mm (white), 50 μm (green).

**Figure 6:**
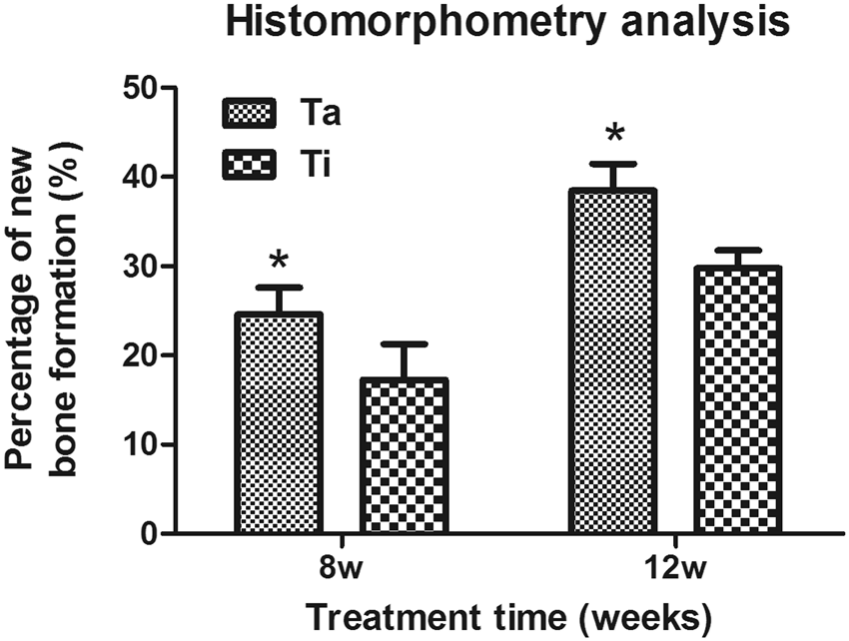
Histomorphometric analysis of Ta-coated TEAVBs and uncoated TEAVBs in defects in the L6 of rabbits. All data are expressed as the mean ± SD. *P < 0.05.

The Von-Gieson (VG) staining showed that at the cell culture time of 8 w, there was a large amount of newly formed bone on the interface between Ta-coated TEAVBs and the residual vertebral bony substance, and some grew into the scaffolds, showing significantly higher ossification effects than those with the uncoated TEAVBs. Although there was some new bone on the uncoated TEAVBs, most was in the form of spongy bone bridges, without apparent calluses, masses, or ingrowth into the scaffolds (Fig. 5A,B,E,F). At 12 w, a large amount of bone was newly formed on the interface of and inside the scaffolds. Moreover, the newly formed bone tissues had bone cells and bound well to the scaffolds, and the interface was partially fused. Regarding the uncoated TEAVBs, however, despite some newly formed bone on the interface and inside the scaffolds, the amount was small, and many parts of the internal scaffolds were still filled by microscopic tissues (Fig. 5C,D,G,H). In addition, the histomorphometry analysis showed that, compared with Ti TEAVBs, the Ta-coated TEAVBs had better bone formation at both 8 and 12 w (P < 0.01) (Fig. 6).

### Biomechanical examination

**Figure 7:**
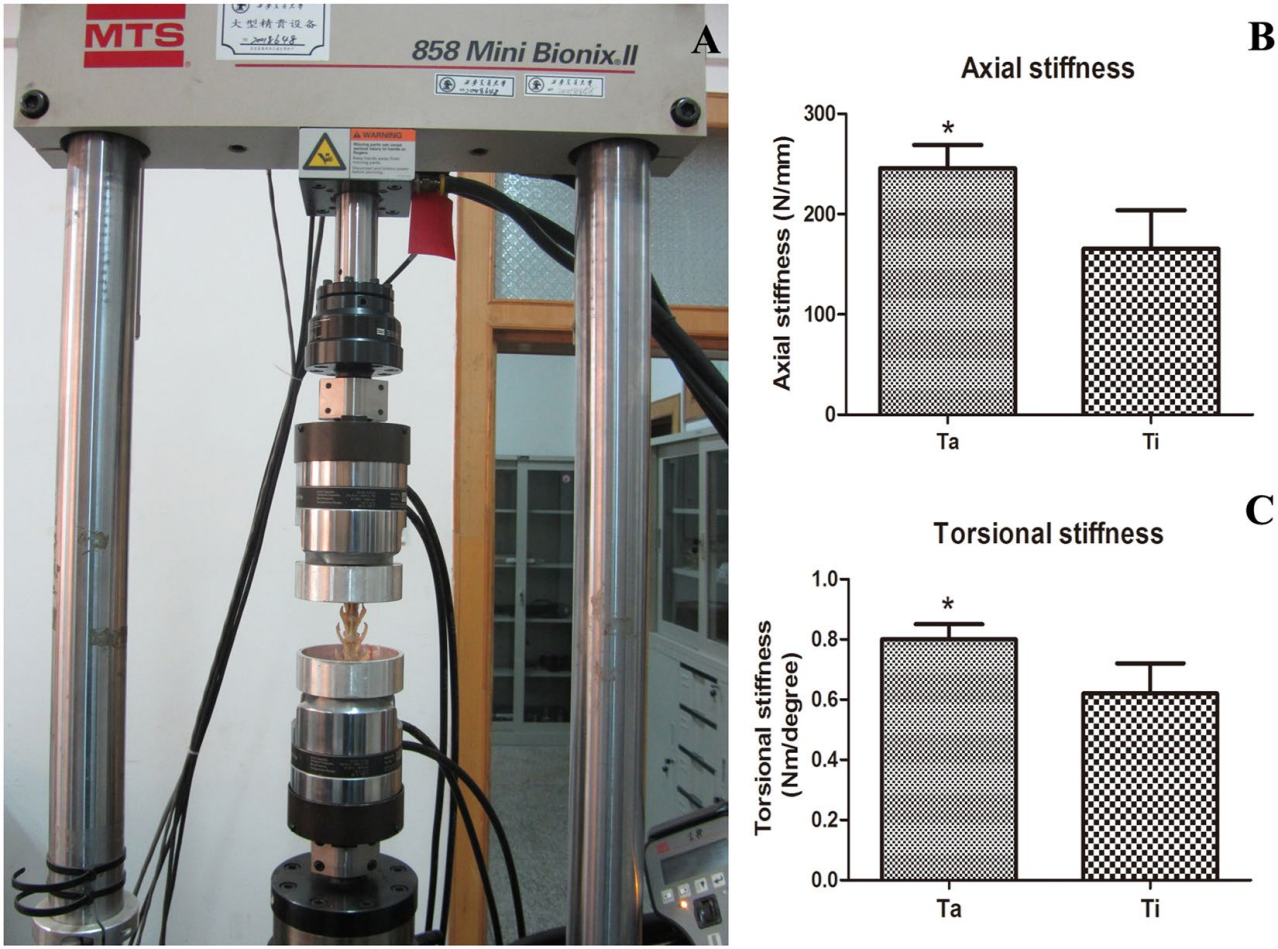
Biomechanical evaluation of scaffolds. (**A**) Overview of the biomechanical testing process. (**B**) Axial stiffness and (**C**) torsional stiffness of the TEAVBs constructed from uncoated and Ta-coated scaffolds at 12 w postoperation. All data are expressed as the mean ± SD. *P < 0.05.

At the cell culture time of 12 w, the specimens were measured for vertical and rotational stiffness. As a result, TEAVBs constructed from Ta-coated scaffolds had vertical and rotational stiffnesses that were significantly different from those of TEAVBs constructed from Ti scaffolds (P < 0.05). The results showed that, compared with the uncoated TEAVBs, when the Ta-coated TEAVBs were used to repair rabbit lumbar vertebral defects, the ability to withstand deformation was enhanced (Fig. 7B,C).

## Discussion

Ti cages and AVBs are widely used in the clinic. Despite many advantages, their defects gradually appear. In particular, most Ti cages and AVBs are composed of Ti-6A1-4V, which has no osteoconductive, osteoinductive or osteogenic effects. Therefore, many scientists have used different methods to modify their osteogenic characteristics, and a porous Ti-6A1-4V scaffold vector compounded with BMSCs in vertebral defect repair solves these problems. The porous structure of the Ta-coated Ti-6A1-4V scaffold provides a place for vascular ingrowth and new bone formation, which presents osteoconductivity. Due to its high strength, corrosion resistance and heat resistance, Ti-6A1-4V is widely used in orthopedic implants and porous scaffolds to construct extraneous tissue-engineered bones and has yielded good results^29^. However, the adhesion and proliferation of osteoblasts on the surface of porous Ti-6A1-4V scaffolds are unsatisfactory. Along with the diversification of material surface treatment methods and the wide application of Ta materials in orthopedic surgery, Ta-compound materials have stood out as bone implants, which provides a theoretical basis for the application of Ta materials in orthopedic surgery^30,31^.

Orthopedic implants made from traditional metal materials have no bioactivity and cannot become bones. To enhance their bioactivity, some scholars have used various bioactive coatings. The existing literature on orthopedics, craniofacial science and dentistry shows that Ta implants have outstanding biocompatibility and security^18^. The basic structure of porous Ta features high porosity, a low elastic modulus and relatively high friction. Ta has high corrosion resistance in highly acidic environments and does not change in weight or surface roughness, in contrast to Ti and stainless-steel implants^32^. The overall corrosion resistance of porous Ta may be associated with its small stress shield and high load-bearing capacity. These intrinsic properties and its high biocompatibility endow porous Ta with optimized design and use properties. Currently, porous Ta is widely used in orthopedic implants in arthroplasty prostheses, bone graft replacements, and cartilage scaffolds.

Most of the literature has focused on pure Ta or Ta alloy implants, which are expensive and unfavorable for wide application. When the Ta coating is plasma-sprayed on the surface of other metals, the biocompatibility of the metal implants can be enhanced. However, there are few studies on the coating of Ta onto extraneous porous Ti-6A1-4V scaffolds to construct tissue-engineered bone. When Ta-coated Ti-6A1-4V was used to repair bone defects *in vitro*, the Ta coating improved cell proliferation and the adhesion of human osteoblasts on the surface of scaffolds, providing a biological basis for the surface modification of biomaterials^33^. However, there are few studies on the effects of Ta-coated Ti-6A1-4V scaffold vectors in bone defect repair *in vivo* or on the application of Ta-coated Ti-6A1-4V scaffolds for the repair of vertebral defects.

Ta and Ti have slightly different biocompatibilities in terms of interface and tissue reaction^34^. *In vivo* histological studies have shown polykaryocytes around Ta implants but not Ti implants. When Ta wire was implanted into rat soft tissues and femurs to observe the reactions of soft and hard tissues^23^, the area around the Ta implants had no inflammatory reaction but high corrosion resistance. Furthermore, Ta did not degrade after 4 weeks, showing high biocompatibility. Other histological studies showed that the surrounding soft tissues had no sign of inflammatory reaction or polykaryocytes^20,22,35,36^. These results demonstrate that the coating of Ta onto porous Ti-6A1-4V scaffolds will improve biocompatibility, and these scaffolds provide a basis for future cytology experiments and *in vivo* animal experiments.

There are no clinical animal models on the application of TEAVB to the repair of vertebral defects. The rabbit lumbar vertebral corpectomy model is simple, convenient, and easy to perform compared with dog and calf models^27,37,38^. This model has not been reported elsewhere. Shirado *et al*.^39^ used a biodegradable polymer to repair dog lumbar anterior vertebra subtotal corpectomy. They found that this polymer was not associated with any obvious host bone rejection and ossification was favorable during the 6-month follow-up, but the mechanical strength was low, which may cause partial spinal instability. Itoh *et al*.^40^ used HA-mixed collagen fiber and BMP to make tissue-engineered bones and use them to repair dog lumbar defects. At 13 weeks, the materials were basically absorbed; abundant spongy bone bridges were formed between materials and the remaining vertebral bony substance, and new bone had formed. They recommended HA-mixed collagen fiber and BMP for the repair of vertebral defects.

Findlay and Miyazaki^25,41^ compared the effects between flat-disc-shaped porous Ta implants and traditional implants in the repair of human osteoblasts and found no obvious difference in cell growth rate, cell adhesion, gene expression, cell morphology, bone mineralization *in vitro*, or mRNA expression levels of genes involved in bone damage and bone formation. They concluded that porous Ta is an ideal material that promotes the growth and differentiation of human osteoblasts. In our study, after BMSCs inoculated with scaffold vectors were cultured *in vitro* for 72 h, the BMSCs growing on the Ta-coated porous Ti-6A1-4V scaffolds had better proliferation and adhesion, and their pseudopods adhered more closely to the surface of scaffolds compared with BMSCs growing on uncoated scaffolds, indicating the Ta-coated porous Ti-6A1-4V scaffolds had higher biocompatibility and were favorable for cell adhesion and proliferation.

Our study confirmed that Ta-coated porous Ti-6A1-4V scaffolds compounded with BMSCs had increased osteogenic activity, demonstrating successful osteoconductivity, osteoinductivity and osteogenesis and making this scaffold material favorable for the ingrowth of the surrounding newly formed bone tissues and for the bone fusion at sites near vertebral defects. From porous Ti-6A1-4V scaffolds, to Ta-coated porous Ti-6A1-4V scaffolds to Ta-coated TEAVBs, we successfully constructed active AVBs. The favorable mechanical performance, tissue compatibility, and joint effects of osteoconductivity, osteoinductivity and osteogenesis may make it possible to use TEAVBs in the repair of vertebral defects.

## Materials and Methods

### Scaffold preparation and surface characterization

The porous Ti scaffold with a diameter of 5 mm and a height of 24 mm was designed using commercial computer-aided design (CAD) software and produced on an Arcam’s EBM machine (EBM A1; Arcam AB, Sweden) as previously described^16^. Ta coating on the porous Ti scaffold was performed with a chemical vapor deposition (CVD) method as reported by Levesque and Li^28,42^. After the CVD process, all the scaffolds were ultrasonically cleaned in methanol and then rinsed with distilled water (Fig. 1A). All the Ti and Ta-coated scaffolds were characterized by microcomputerized tomography (micro-CT, eXplore Locus SP, GE Healthcare, Canada) (Fig. 1B) and scanning electron microscopy (SEM, S-3400, HITACHI, Japan) (Fig. 1C,D).

### *In vitro* study

#### Culture of rabbit BMSCs

Primary rabbit BMSCs were obtained by flushing the femur and tibiae of 200 g, 7-day-old New Zealand rabbits with phosphate-buffered saline (PBS), after which they were filtered through a strainer and suspended in Dulbecco’s Modified Eagle’s Medium (DMEM, Sigma) supplemented with 10% fetal bovine serum (FBS, Gibco) and 1% penicillin/streptomycin^1^. Then, the cells were incubated in a 5% humidified CO_2_ chamber at 37 °C. When the cells reached 90% confluence, they were trypsinized with 0.25% trypsin for 1 min at 37 °C. Cells were passaged once per week, and the third-passage BMSCs were seeded on Ti and Ta-coated scaffolds at a density of 5 × 10^5^ cells/ml. Then, they were cultured in an incubator at 37 °C and 5% humidified CO_2._

#### The observation of cell morphology

Cells were labeled by chloromethyl-benzamidodialkylcarbocyanine (CM-DiI) as previously described^2^. Stock solutions of CM-DiI were prepared in dimethyl sulfoxide (DMSO) at 1 g/L. Immediately before labeling, the 1 g/L stock solution was diluted with PBS to a working concentration of 2 mg/L. The third-passage BMSCs were digested into cell suspensions, and 1 mL of cell suspension was added and fully mixed with 10 μl of DiI solution, followed by thermostatic incubation in a cell incubator at 37 °C and 5% CO_2_ for 30 min. Then, the cell suspension was taken out and centrifuged at 1200 rpm for 5 min. The supernatant was removed, and the cell suspension was put into DMEM supplemented with FBS for centrifugation, which was repeated 3 times. After cell labeling, the BMSCs were seeded on Ti and Ta-coated scaffolds at a density of 5 × 10^5^ cells/ml and were cultured in an incubator at 37 °C and 5% humidified CO_2_. After 72 h of incubation, the scaffolds were taken out, observed under a fluorescence microscope (LEICA TMLA, Germany), and photographed by a cold-light-source digital camera imaging system (PIXERA, USA). Then, all the scaffolds were washed with PBS 3 times and fixed by adding 5 ml of 2% glutaraldehyde, followed by progressive dehydration with anhydrous ethanol, critical-point drying, and gold spraying. The morphology of the scaffolds was characterized by scanning electron microscopy (SEM, S-3400, HITACHI, Japan).

#### Analysis of cell adhesion and proliferation

Cell adhesion was evaluated using methylthiazolyl tetrazolium (MTT). The media were discarded after cell culture for 2, 4 or 8 h, and after washing with PBS 2 times, each implant was added to 200 μl of 5 g/L MTT and 4 ml of 10% DMEM for incubation for 4 h. The precipitated formazan was then dissolved in DMSO. After 10 min of slow shaking, the optical density (OD) was measured at 570 nm by a Bio-Rad 500 spectrophotometric microplate reader. Cell proliferation was assessed by the same method. After cell culture for 1, 4, or 7 d, MTT solution was added, and the OD at 570 nm was measured.

#### Analysis of cell alkaline phosphatase activity

The ALP activities of the samples were determined by a colorimetric assay using an ALP reagent containing p-nitrophenyl phosphate (p-NPP) as the substrate after 7 d of culturing. The culture medium that we used in the ALP assay was Dulbecco’s Modified Eagle’s Medium (DMEM, Sigma) supplemented with 10% fetal bovine serum (FBS, Gibco), 1% penicillin/streptomycin, 2 mM L-glutamine, 100 nM dexamethasone, 50 μM L-ascorbic acid-2-phosphate and 10 mM β-glycerophosphate (Sigma). The absorbance of p-nitrophenol formed was measured at a wavelength of 405 nm. The intracellular total protein content was estimated using a protein assay kit (Pierce, Rockford, IL), and the ALP activity was expressed as μmol/h/mg of protein/g of scaffold.

### *In vivo* study

#### Animals and ethics

Sixteen New Zealand white rabbits (6-month-old males, weighing 3.0 ± 0.5 kg) were acquired following the National Institutes of Health Guidelines for the Use of Laboratory Animals, and all procedures were approved by the Fourth Military Medical University Committee on Animal Care. Before the experiment, a digital X-ray machine (Siemens, Germany) was used to photograph the anterior and lateral lumbar vertebra to exclude adverse factors such as spinal deformity and fracture. A dual-energy X-ray densitometer (Lunar Corp., Madison, WI, USA) under animal implant mode was used to measure the rabbit lumbar vertebral body (L6) BMD to exclude the effects of BMD variation on the subsequent *in vivo* study.

#### Construction of TEAVB

The Ti and Ta-coated scaffolds were prewetted with PBS and placed in 6-well culture plates, and the third-passage BMSCs were seeded on Ti and Ta-coated scaffolds at a density of 5 × 10^5^ cells/ml. After the addition of 5 ml of DMEM supplemented with FBS and 1% penicillin/streptomycin, the seeded scaffolds were cultured in an incubator at 37 °C and 5% humidified CO_2_ for 1 week, and the media were changed every 2 d.

#### Surgical procedures

**Figure 8:**
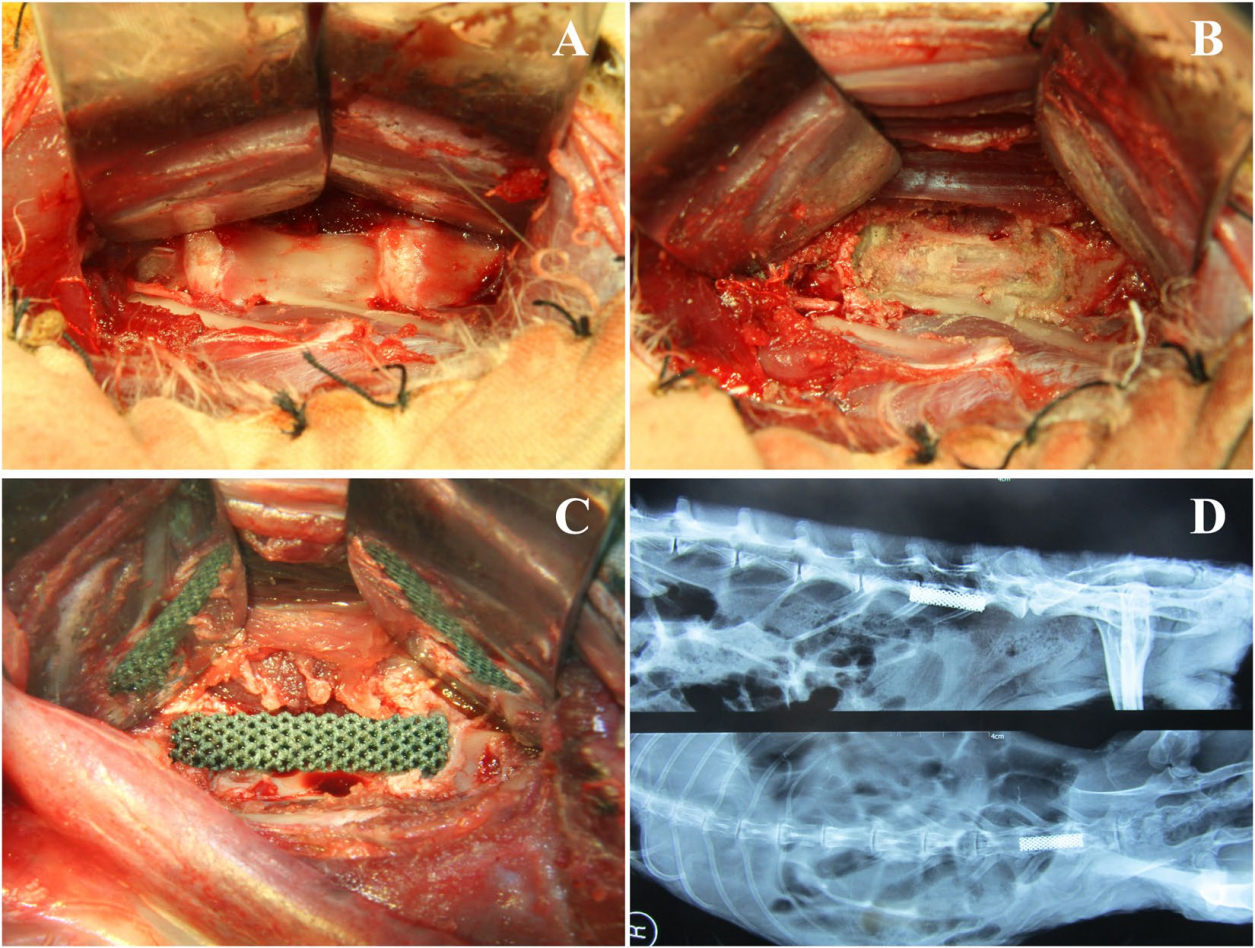
The surgical procedure of the implantation of a Ta-coated scaffold into defects in the L6 of a rabbit. (**A**) The exposure of the forepart of the lumbar vertebral body. (**B**) L6 corpectomy. (**C**) The implantation of the scaffold into the defect site. (**D**) X-ray examination during the operation to check the positions of TEAVBs. Top: lateral radiograph; bottom: anteroposterior image.

All the rabbits were placed in quarantine for 7 d prior to surgery. After general anesthesia with an intramuscular injection of a mixture of diazepam (0.1 mg/kg) and ketamine (2 mg/kg), the rabbit was fixed in the supine position, and the abdomen was shaved. After the skin was sterilized by hydrogen peroxide and iodophor, an 8-cm oblique incision was made in the left abdomen, along the space between the internal oblique muscle of the abdomen and the peritoneum bluntly to the deep left side to expose the left psoas major. Until the left transverse process of the lumbar vertebra was reached, hemostatic forceps were used to bluntly dissect the greater psoas and expose the transverse process of the lumbar vertebrae. Along the transverse process, the vertebral body was bluntly dissected, and the muscles, soft tissues and nerve blood vessels were pushed by using a drag hook to the contralateral side to fully expose the forepart of the L6 lumbar vertebral body (Fig. 8A). Then, L6 corpectomy was performed, including the adjacent upper and lower intervertebral discs, which were excised to expose the endplates of the adjacent upper and lower vertebral bodies, respectively (Fig. 8B). The Ti and Ta-coated TEAVBs were finally implanted into the defect site, and X-ray examination was performed to check the positions of the TEAVBs (Fig. 8C,D). Then, the subcutaneous tissue and skin were closed in different layers. The surgical site was finally covered with an adhesive bandage. After the surgery, cephazolin (10 mg/kg) was routinely administered intramuscularly as a prophylactic antibiotic once a day for 5 d. Eight and 12 weeks after surgery, the rabbits were euthanized, and the specimens containing the implants and surrounding bone tissue were harvested for further examinations.

#### Micro-CT analysis

The specimens were dissected into columns 35 mm long and 15 mm wide, centered around the TEAVB (n = 6 in each group). They were fixed in 10% neutral buffered formalin, placed in the sample holder and scanned using the micro-CT system (eXplore Locus SP, GE Healthcare, USA). The conditions were the following: resolution 21 μm, rotation angle 300°, exposure time 2960 ms, average frame number 6, pixel combination 1 × 1, black and white scanning calibration, increment of rotation angle 0.5°, voltage 80 kV, current 80 μA, and Hounsfield calibration. Specimens were constructed and evaluated using 3D analysis software (MicroView, GE Healthcare, USA). A cylindrical area with a diameter of 3 mm around each TEAVB was selected as the region of interest (ROI); the reconstruction threshold was set at 900; and Advanced Bone Analysis (GE Healthcare, USA) was used for bone tissue metrological analysis.

#### Histological and histomorphometry analysis

All the specimens were fixed in 10% formalin for 3 weeks. After dehydration in a graded ethanol series (70–100%), the specimens were embedded in methylmethacrylate (MMA) solution that polymerized at 37 °C within 1 week until the implants were solidified into blocks. Thin sections of the specimens (100 μm thick) were prepared by a modified interlocked diamond saw (Leica Microtome, Wetzlar, Germany). Then, the sections were stained with 1.2% trinitrophenol and 1% acid fuchsin (Von-Gieson staining) and placed on the sample holder of a standard light microscope (Leica LA Microsystems, Bensheim, Germany) for observation of the growth of bony substance on the TEAVB interface and the surrounding area.

#### Biomechanical testing

At the twelfth week, the lumbar implants were harvested (n = 3 in each group) and fixed by two specially designed mild steel cylinder connectors with dental cement (Meliodent, Heraeus Kulzer, USA) onto an MTS 858 material testing machine (858 Mini-Bionix II, MTS, USA) (Fig. 8A). Nondestructive testing was performed under a 200-N axial load and 15-N·m torsional load^38,43^. The measured parameters from the machine were collected, and the axial and torsional stiffnesses were calculated by relevant software (MTS).

### Statistical analysis

All data were analyzed with the Statistical Package for the Social Sciences (SPSS) for Windows version 15.0 and are presented as the mean value ± standard deviation (SD). Statistical analysis was performed using either one-way analysis of variance (ANOVA) or Student’s t-test. Values of P < 0.05 were considered statistically significant.
